# Burden of respiratory tract infections at post mortem in Zambian children

**DOI:** 10.1186/s12916-016-0645-z

**Published:** 2016-07-01

**Authors:** Matthew Bates, Aaron Shibemba, Victor Mudenda, Charles Chimoga, John Tembo, Mwila Kabwe, Moses Chilufya, Michael Hoelscher, Markus Maeurer, Sylvester Sinyangwe, Peter Mwaba, Nathan Kapata, Alimuddin Zumla

**Affiliations:** HerpeZ, University Teaching Hospital, Lusaka, Zambia; University of Zambia and University College London Medical School (UNZA-UCLMS) Research and Training Programme, University Teaching Hospital, Lusaka, Zambia; Department of Pathology & Microbiology, University Teaching Hospital, Lusaka, Zambia; Institute for Infectious Diseases, Tongji Medical College, Huazhong University of Science and Technology, Wuhan, China; Division of Infectious Diseases and Tropical Medicine, Medical Centre of the University of Munich, Munich, Germany; Therapeutic Immunology, Department of Laboratory Medicine, Department of Microbiology, and Department of Tumour and Cell Biology, Karolinska Institute, Stockholm, Sweden; Department of Paediatrics & Child Health, University Teaching Hospital, Lusaka, Zambia; Ministry of Health, Lusaka, Zambia; National Tuberculosis Control Programme, Ministry of Community Development, Maternal and Child Health, Lusaka, Zambia; Department of Infection, Division of Infection and Immunity, University College London, and NIHR Biomedical Research centre at UCL Hospitals, London, UK

**Keywords:** Autopsy, Post mortem, Children, Zambia, Africa, Tuberculosis, Pneumonia, Cytomegalovirus, *Pneumocystis Jirovecii* pneumonia

## Abstract

**Background:**

Autopsy studies are the gold standard for determining cause-of-death and can inform on improved diagnostic strategies and algorithms to improve patient care. We conducted a cross-sectional observational autopsy study to describe the burden of respiratory tract infections in inpatient children who died at the University Teaching Hospital in Lusaka, Zambia.

**Methods:**

Gross pathology was recorded and lung tissue was analysed by histopathology and molecular diagnostics. Recruitment bias was estimated by comparing recruited and non-recruited cases.

**Results:**

Of 121 children autopsied, 64 % were male, median age was 19 months (IQR, 12–45 months). HIV status was available for 97 children, of whom 34 % were HIV infected. Lung pathology was observed in 92 % of cases. Bacterial bronchopneumonia was the most common pathology (50 %) undiagnosed ante-mortem in 69 % of cases. Other pathologies included interstitial pneumonitis (17 %), tuberculosis (TB; 8 %), cytomegalovirus pneumonia (7 %) and *pneumocystis Jirovecii* pneumonia (5 %). Comorbidity between lung pathology and other communicable and non-communicable diseases was observed in 80 % of cases. Lung tissue from 70 % of TB cases was positive for *Mycobacterium tuberculosis* by molecular diagnostic tests. A total of 80 % of TB cases were comorbid with malnutrition and only 10 % of TB cases were on anti-TB therapy when they died.

**Conclusions:**

More proactive testing for bacterial pneumonia and TB in paediatric inpatient settings is needed.

## Background

Global Burden of Disease study estimates suggest that, for children, bacterial pneumonia is the leading single cause of death, responsible for 23 % of deaths in children aged between 27 days and 5 years of age [1]. Respiratory pathology may also play a role in additional deaths, as bacterial, fungal or viral lung infections may underlie other major causes of death, including infections such as malaria or diarrhoeal disease, as well as non-communicable diseases such as malnutrition [2–4]. Determining the aetiology of childhood respiratory deaths in the African context is particularly challenging – symptoms are non-specific, obtaining specimens for microbiological analysis from infants and young children ante mortem is difficult, and in the low-resource settings laboratory services cannot provide a thorough microbiological work-up combining culture with the latest multiplex molecular diagnostics [4, 5]. It is hence extremely difficult for the attending physician to differentiate between bacterial, mycobacterial, fungal or viral aetiologies [6, 7]. The non-specific symptoms of respiratory infections also contribute to the inaccuracy of verbal autopsy studies, which rely on interviewing relatives or the attending physician [8].

The gold-standard for determining cause-of-death is anatomical post mortem followed by histopathological examination of selected tissues [9]. Post mortem studies have been rarely undertaken in the African context because they are expensive and difficult to implement, requiring highly skilled personnel and sophisticated infrastructure, and because they are culturally unpalatable, particularly with respect to children [4, 10, 11]. Furthermore, the results of autopsy studies are often overlooked by epidemiologists due to the relatively small sample sizes compared to larger and easier to implement surveys of clinical records and verbal autopsy studies. However, when autopsy studies have been undertaken, they often yield surprising results. In 2002, we conducted a landmark autopsy study of 264 Zambian paediatric deaths [12], which influenced World Health Organization policy with respect to the burden of paediatric tuberculosis (TB), and led to studies to treat *Pneumocystis Jirovecii* pneumonia (PCP) in HIV-infected children [13].

The decade that followed has seen the roll out of anti-retroviral therapy (ART) and prevention of mother-to-child transmission programmes. We conducted a prospective autopsy study to describe the histopathological and microbiological findings derived from the examination of lungs at post mortem among inpatient children who died at the University Teaching Hospital (UTH), Lusaka, Zambia.

## Methods

### Study design

We undertook a cross-sectional autopsy study of inpatient paediatric deaths at UTH, Zambia’s national referral centre, to determine the burden of respiratory pathology among children dying at the hospital. All children < 15 years of age who died in the inpatient wards at UTH were eligible for inclusion in the study. Necropsy restricted to the chest cavity was performed. Autopsy findings and outcome data on respiratory causes-of-death were compared with the cause-of-death given by the attending physician. Baseline age and sex of all inpatient paediatric deaths during the recruitment period was extracted from hospital mortality records to allow a rough estimate of how the study group might be representative of all paediatric mortalities within the hospital.

### Recruitment and consent

The recruitment process takes several hours and involves counselling the relatives and talking about the child who has died, before then introducing the idea of the autopsy investigation and explaining the purpose and rationale of the study. Due to time constraints, it was not possible to approach the relatives of all children who died during the study period. The recruiting clinical officer (CC) worked Monday to Friday and so the relatives of children who died between Friday afternoon and Sunday morning were unlikely to be approached to take part in the study. In Zambia, there is a cultural requirement that there be minimal delay in burying children. After being alerted to deaths by the attending physician, our multi-lingual clinical officer would approach the relatives to offer counselling, introducing them to the study in their native language and providing them with written information sheets (available in English, Chi-Nyanja and Chi-Bemba) explaining the purpose of the study. The attending relatives were given the opportunity to consult with family elders and to ask any questions they might have, and reasons for refusing consent were recorded. Consenting families were given a payment of $10 to compensate for the delayed release of the deceased for burial. The HIV status of the children in this study is taken from either neonatal PCR testing (in children under 18 months of age) and/or standard serology testing in accordance with Zambian national guidelines in children aged 18 months and older. Ethical approval for the study was granted by the University of Zambia Biomedical Research Ethics Committee.

### Autopsy examination and sampling

One of two consultant pathologists (VM and AS) undertook a limited necropsy examination within 18 hours after death, examining the lungs, intrathoracic lymph nodes, heart, kidneys, pancreas, spleen and liver. Gross pathology was recorded and photographed, organs weighed and dissected, with lung samples taken for histopathology and aseptically for cryopreservation (stored at −80 °C), employing strict safety procedures as previously described [14]. Samples were obtained from all five lobes guided by gross pathology. In the absence of gross pathology a representative specimen was sampled.

### Histopathology

Histopathologists, blinded to clinical data, performed haematoxylin and eosin, silver methenamine, periodic acid-Schiff and Zeihl–Neelsen stains as previously described [14]. Pathological findings were defined as described [14, 15]. Post mortem diagnoses were based on a composite of gross pathology and histopathology. Cultures were not undertaken as the UTH TB lab had insufficient capacity to culture TB from tissue specimens within the timeframe of study commencement, and also because a negative culture does not exclude TB disease at autopsy. Zeihl–Neelsen stains were undertaken on all suspected TB cases.

### Molecular analysis

Cryopreserved lung tissue was analysed with the Xpert MTB/RIF assay as previously described [14]. This cartridge based molecular diagnostic test detects *Mycobacterium tuberculosis* (*M.tb*) complex DNA and rifampicin resistance, a reasonable proxy marker of multi-drug resistant-TB [16, 17]. For non-TB mycobacteria (NTM) analysis, DNA was extracted from cryopreserved lung tissue using the ‘E0101 DNA extraction kit’, and screened with the ‘PowerChek™ MTB/NTM Real-time PCR Kit’ (both supplied by Kogene, South Korea), in accordance with manufacturer's instructions. This internally controlled Real-Time PCR assay uses a generic pan-mycobacteria probe (target 16S‐23S rRNA internal transcribed spacer (ITS) region) and an *M.tb* specific probe(target IS6110) to differentiate *M.tb* from NTM.

### Data management and statistical analysis

We undertook double-data entry using Epidata and exported cleaned datasets for analysis in SPSS version 21. Point prevalences were weighted based on the age distribution and sex of the population of deaths at the hospital as previously described [14].

## Results

### Case recruitment and descriptives

**Fig. 1:**
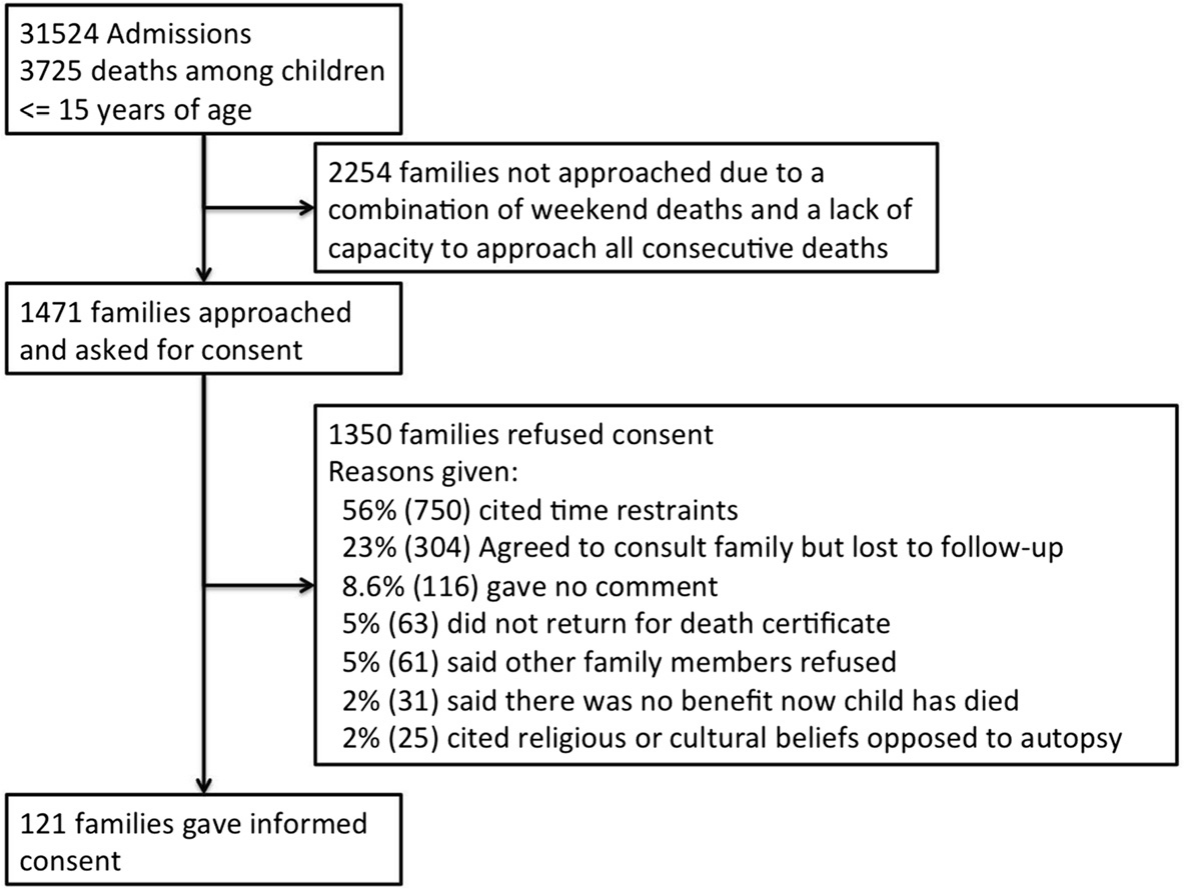
Recruitment flow diagram

During the study period (August 2011 to June 2014) there were a total of 3725 deaths among paediatric admissions under 15 years of age. The recruiting clinical officer approached 1471 families and obtained consent for participation in the study from 121 families (Fig. 1). The main reasons for refusing consent were time constraints on taking the child for burial and loss to follow-up after agreeing to consult with family (Fig. 1). Of 121 children autopsied, 60 % (73/121) of cases were male, median age was 19 months (IQR, 12–45 months), and 35 % (34/96) of children were HIV infected (Table 1). ART status was determined for 24 cases, of which 63 % (15) were on ART when they died. Overall, 12 % (15/121) of children were receiving anti-TB therapy. Comparing age and sex between study and non-study mortalities, we found that the age distribution of participants in our study was significantly older and that the percentage of male children was higher (60 %, 73/121) than among non-study deaths (50 %, 1810/3604; *P* = 0.029; Table 1). The median duration of stay in hospital was 2 days (IQR, 1–6 days).

**Table 1:** Comparison of age and sex distribution between study and population deaths

	Population (*n* = 3604)^a^	Study (*n* = 121)	*P* ^b^
Age (months)			
Median (IQR)	NA	19 (12–45)	NA
<1 years	48.7 % (1787)	29 % (35)	<0.001
1–4 years	36.7 % (1321)	48 % (58)	
4–15 years	13.8 % (496)	23 % (28)	
Male sex	50.2 % (1810)	64 % (77)	0.005
HIV infected	39.4 % (85/216)^c^	34 % (33/97)^d^	0.368

^a^All inpatient deaths that did not take part in the study

^b^χ^2^ test

^c^HIV status of population estimated based on previous study [16]

^d^HIV status unavailable for 24 cases

### Lung pathologies and stratification by HIV status

Lung pathology was observed in 92 % (111/121) of cases (Table 2). Bronchopneumonia was the most common finding, diagnosed in 50 % (60/121) of cases, followed by interstitial pneumonitis in 17 % (20/121). TB was detected in 8 % (10/121) of all deaths (9 pulmonary and 1 extrapulmonary), and was two-fold more prevalent in HIV-infected children, although within the limits of the sample this was not significant (*P* = 0.15). Furthermore, infections that are commonly associated with HIV, such as cytomegalovirus pneumonia, *pneumocystis Jirovecii* pneumonia and candidiasis, were not more prevalent among HIV-infected children, within the limits of the available sample (Table 2). Adjusting for recruitment bias did not significantly affect the distribution of lung pathologies (Table 2).

**Table 2:** Lung pathology findings

	Non-weighted				Weighted for age and sex
	Overall	HIV uninfected	HIV Infected	HIV status unknown	Overall
	(*n* = 121)	(*n* = 62)	(*n* = 34)	(*n* = 25)	(*n* = 121)
	Count (%)	Count (%)	Count (%)	Count (%)	Count (%, SE)
Lung pathology	111 (92 %)	53 (86 %)	34 (100 %)	24 (96 %)	112 (93 %, 2.4)
Bronchopneumonia	60 (50 %)	28 (45 %)	16 (47 %)	16 (64 %)	52 (43 %, 4.5)
Interstitial pneumonitis	20 (17 %)	9 (15 %)	7 (21 %)	4 (16 %)	21 (17 %, 3.4)
Tuberculosis (All forms) (PTB and/or EPTB)	10 (8 %)	4 (6 %)	5 (15 %)	1 (4 %)	11 (9 %, 2.6)
EPTB	1 (1 %)	0 (0 %)	1 (3 %)	0 (0 %)	1 (1 %, 0.8)
PTB	9 (7 %)	4 (6 %)	4 (12 %)	0 (0 %)	10 (8 %, 2.5)
Cytomegalovirus pneumonia	8 (7 %)	4 (6 %)	3 (9 %)	1 (4 %)	10 (9 %, 2.5)
*Pneumocystis Jirovecii* Pneumonia	6 (5 %)	2 (3 %)	3 (9 %)	1 (4 %)	13 (11 %, 2.9)
Pulmonary oedema	6 (5 %)	5 (8 %)	0 (0 %)	1 (4 %)	4 (4 %. 1.7)
Candidiasis	3 2 %)	3 (5 %)	0 (0 %)	0 (0 %)	1 (1 %, 0.9)
Pleuritis	2 (2 %)	1 (2 %)	0 (0 %)	1 (4 %)	1 (1 %, 0.8)
Lymphoid interstitial pneumonitis	2 (2 %)	0 (0 %)	1 (3 %)	1 (4 %)	1 (1 %, 0.7)
Lobar pneumonia	1 (1 %)	0 (0 %)	1 (3 %)	0 (0 %)	1 (1 %, 0.8)
Acute respiratory distress	1 (1 %)	1 (2 %)	0 (0 %)	0 (0 %)	3 (3 %, 1.5)
Pulmonary haemorrhage	1 (1 %)	1 (2 %)	0 (0 %)	0 (0 %)	1 (1 %, 0.7)
Normal lungs	10 (8 %)	9 (15 %)	0 (0 %)	1 (4 %)	9 (8 %, 2.4)

*EPTB* extrapulmonary tuberculosis, *PTB* pulmonary tuberculosis

### Comparison between lung pathology and diagnosis given by the attending physician

We identified comorbidity between two or more communicable and non-communicable diseases in 80 % (97/121) of cases (Table 3). Malnutrition (defined broadly as a composite of Kwashiorkor/Protein Energy Malnutrition or Marasmus) was the major co-morbidity with 93 % (57/61) of malnourished children having a comorbidity. Among non-malnourished children, comorbidity was observed in 67 % (40/60) of cases (Table 3). Among the 111 children with lung pathology, the diagnoses given by the attending physician indicated a range of possible comorbid conditions, with both communicable and non-communicable diseases (Table 3). Malnutrition was the predominant comorbidity, present in 50 % (56/111) of cases with lung pathology, and was the predominant comorbidity for the five most prevalent lung pathologies: bronchopneumonia, interstitial pneumonitis, TB, CMV pneumonia and PCP (Table 3). Lung pathology was observed in 100 % (15/15) of children diagnosed with other non-communicable diseases including cancer, heart disease and developmental disorders (Table 3).

**Table 3:** Comparison between lung pathology and diagnosis given by the attending physician

		Lung pathology from post mortem					
Attending physician diagnosis	No lung pathology	Any lung pathology	Bronchopneumonia	Interstitial pneumonitis	Tuberculosis	CMV pneumonia	PCP
	(*n* = 10)	(*n* = 111)	(*n* = 59)	(*n* = 20)	(*n*=10)	(*n* = 8)	(*n* = 6)
Communicable diseases^a^							
Pneumonia	20 % (2/10)	30 % (33/111)	31 % (18/59)	15 % (3/20)	40 % (4/10)	50 % (4/8)	50 % (3/6)
Sepsis/septic shock	40 % (4/10)	23 % (25/111)	22 % (13/59)	15 % (3/20)	20 % (2/10)	50 % (4/8)	17 % (1/6)
Acute diarrhoea and dehydration	40 % (4/10	23 % (26/111)	27 % (16/59)	35 % (7/20)	0 % (0/10)	25 % (2/8)	0 % (0/6)
Meningitis	30 % (3/10)	14 % (15/111)	8 % (5/59)	15 % (3/20)	30 % (3/10)	0 %(0/8)	17 % (1/6)
Tuberculosis	0 % (0/10)	8 % (9/111)	3 % (2/59)	5 % (1/20)	40 % (4/10)	13 % (1/8)	17 % (1/6)
Malaria	0 % (0/10)	5 % (5/111)	3 % (2/59)	15 % (3/20)	0 % (0/10)	0 % (0/8)	0 % (0/6)
Typhoid/enteric fever	10 % (1/10)	3 % (3/111)	2 % (1/59)	5 % (1/20)	0 % (0/10)	0 % (0/8)	17 % (1/6)
PCP	0 % (0/10)	3 % (3/111)	3 % (2/59)	0 % (0/20)	0 % (0/10)	25 % (2/8)	17 % (1/6)
Hepatitis	0 % (0/10)	1 % (1/111)	2 % (1/59)	0 % (0/20)	0 % (0/10)	0 % (0/8)	0 % (0/6)
Tetanus	0 % (0/10)	1 % (1/111)	2 % (1/59)	0 % (0/20)	0 % (0/10)	0 % (0/8)	0 % (0/6)
Non-communicable diseases^b^							
Kwashiorkor/PEM, Marasmus or PCM	40 % (4/10)	50 % (56/111)	53 % (31/59)	55 % (11/20)	80 % (8/10)	63 % (5/8)	33 % (2/6)
WAZ score < −2.0 (children aged < 10 years old)	78 % (7/9)	63 % (57/91)	62 % (31/50)	56 % (9/16)	75 % (6/8)	63 % (5/8)	75 % (3/4)
Non-malnutrition	0 % (0/10)	14 % (15/111)	15 % (9/59)	10 % (2/20)	10 % (1/10)	0 % (0/8)	17 % (1/6)
Leukaemia	0 % (0/10)	1 % (1/111)	2 % (1/59)	0 % (0/20)	0 % (0/10)	0 % (0/8)	0 % (0/6)
Kaposi’s sarcoma	0 % (0/10)	2 % (2/111)	2 % (1/59)	0 % (0/20)	10 % (1/10)	0 % (0/8)	0 % (0/6)
Cerebral palsy	0 % (0/10)	3 % (3/111)	3 % (2/59)	5 % (1/20)	0 % (0/10)	0 % (0/8)	0 % (0/6)
Congestive cardiac failure	0 % (0/10)	3 % (3/111)	3 % (2/59)	0 % (0/20)	0 % (0/10)	0 % (0/8)	17 % (1/6)
Rheumatic heart disease	0 % (0/10)	2 % (2/111)	3 % (2/59)	0 % (0/20)	0 % (0/10)	0 % (0/8)	0 % (0/6)
Sickle cell anaemia associated cardiovascular accident	0 % (0/10)	1 % (1/111)	2 % (1/59)	0 % (0/20)	0 % (0/10)	0 % (0/8)	0 % (0/6)
Primary immune deficiency	0 % (0/10)	1 % (1/111)	0 % (0/59)	5 % (1/20)	0 % (0/10)	0 % (0/8)	0 % (0/6)

Percentages indicate proportion of given lung pathology (columns) for which attending physician ascribed stated causes-of-death (rows)

^a^Other infectious causes-of-death without lung pathology: rabies (*n* = 1)

^b^Non-communicable disease causes-of-death with other lung pathologies not tabulated: Burkett’s lymphoma with pulmonary haemorrhage and pleural inflammation (*n* = 1), hepatocellular carcinoma with pulmonary oedema (*n* = 1). Note: Causes-of-death and lung pathologies are not mutually exclusive

*CMV* cytomegalovirus, *PCP pneumocystis Jirovecii* pneumonia, *PCM* protein-calorie malnutrition, *PEM* protein energy malnutrition

Among 59 children diagnosed with histopathologically confirmed pneumonia, only 31 % (18/59) were diagnosed as having pneumonia ante mortem. Other common ante mortem diagnoses among histopathologically confirmed pneumonia cases included sepsis (22 %, 13/59) and acute diarrhoea and dehydration (27 %, 16/59; Table 3). Interstitial pneumonitis presented with a range of comorbidities, including acute diarrhoea and dehydration (35 %, 7/20), pneumonia, sepsis, meningitis and malaria (each 15 %, 3/20). Of 10 cases of histopathologically confirmed TB infection, just 40 % (4/10) were diagnosed ante mortem (Table 3), with only one case having initiated anti-TB therapy before they died (Table 4). The other comorbid diagnoses with confirmed TB were pneumonia (40 %, 4/10), sepsis (20 %, 2/10) and meningitis (30 %, 3/10) (Table 3). CMV pneumonia was diagnosed in cases with gastrointestinal symptoms, and PCP was diagnosed in cases with both gastrointestinal or central nervous system symptoms, in addition to respiratory symptoms (Table 3). Of four children on anti-TB therapy when they died, none had TB pathology detected at post mortem.

**Table 4:** Molecular analysis of lung tissue from 10 histopathologically confirmed TB cases using Xpert MTB/RIF and PowerChek™ MTB/NTM Real-Time PCR assays

ID	Age	Sex	HIV	ATT	ART	ZN	Xpert MTB/RIF Assay	PowerChek™ MTB/NTM Assay
4B072	3 yr	Female	Positive	No	Yes	Negative	MTB (RIF sens)	MTB
4B083	3 yr, 5 m	Male	Positive	No	Yes	Positive	MTB (RIF sens)	MTB
4B008	2 yr, 7 m	Female	Negative	No	No	Positive	MTB (RIF sens)	Invalid
4B036	10 yr	Male	Negative	No	No	Negative	Negative	NTM
4B120	8 yr	Female	Positive	No	Yes	Positive	Negative	NTM
4B055	1 yr, 5 m	Male	Negative	No	No	Negative	Negative	MTB
4B064	10 yr	Male	Positive	No	Yes	Positive	Negative	MTB
4B085	1 yr, 4 m	Male	Positive	Yes	Yes	Negative	Negative	MTB
4B117	5 m	Male	Negative	No	No	Negative	Negative	MTB
4B076	14 yr	Female	Unknown	No	No	Negative	Negative	Negative

*ATT* anti-tuberculosis therapy, *ART* anti-retroviral therapy, *MTB* Mycobacterium tuberculosis, *NTM* non-TB mycobacteria, *ZN* Zeihl–Neelsen staining

### Molecular analysis of lung tissue

The Xpert MTB/RIF assay detected rifampicin-sensitive TB in 30 % (3/10) of lung tissue specimens in which there was histopathological evidence of pulmonary TB infection (Table 4). In addition, the Xpert MTB/RIF assay was positive in 30 % (33/111) of cases in which there was no histopathological evidence of TB infection, including three cases of bronchopneumonia in which rifampicin resistance was detected (Table 5). The ‘PowerChek™ MTB/NTM Real-time PCR assay’ (Kogene, South Korea) detected MTB in 6/10 (60 %) and NTM in 2/10 (20 %) of histopathologically confirmed TB cases. Of the remaining two cases one was negative and the other flagged as invalid (Table 4). Among non-TB cases, the Kogene assay was positive for MTB in 6/111 (5 %) and positive for NTM in 34/95 (39 %) cases, with 15 negatives (14 %) and 47 invalids (47 %). Running all invalid samples again resulted in just one additional NTM positive result. Comparing results of TB molecular analysis with lung pathology showed that molecular evidence of TB infection was readily detectable in a range of cases with different pulmonary pathologies (Table 5).

**Table 5:** Prevalence of MTB detection by both Xpert MTB/RIF and PowerChek™ MTB/NTM Real-Time PCR assays, within groups with specific lung pathologies

	Xpert MTB/RIF Assay	PowerChek™ MTB/NTM Assay^a^	
	MTB detected	MTB detected	NTM detected
Overall	30 % (36/121)	16 % (12/73)	62 % (45/73)
Lung pathology			
Bronchopneumonia	30 % (18^b^/60)	9 % (3/35)	63 % (22/35)
Interstitial pneumonitis	60 % (8/20)	17 % (2/12)	67 % (8/12)
Tuberculosis (All forms) (PTB and/or EPTB)	30 % (3/10)	67 % (6/9)	22 % (2/9)
EPTB	100 % (1/1)	100 % (1/1)	0 % (0/1)
PTB	22 % (2/9)	63 % (5/8)	20 % (2/8)
Cytomegalovirus pneumonia	13 % (1/8)	0 % (0/4)	75 % (3/4)
*Pneumocystis Jirovecii* pneumonia	17 % (1/5)	0 % (0/3)	67 % (2/3)
Pulmonary oedema	33 % (2/6)	20 % (1/5)	80 % (4/5)
Candidiasis	0 % (0/3)	0 % (0/2)	50 % (1/2)
Acute respiratory distress	0 % (0/1)	0 % (0/1)	100 % (1/1)
Normal lungs	20 % (2/10)	0 % (0/7)	72 % (5/7)

^a^The PowerChek™ MTB/NTM Assay gave an ‘invalid’ result in 48 lung tissue specimens, even after repeat analysis

^b^Including three rifampicin-resistant cases

*EPTB* extrapulmonary tuberculosis, *MTB* Mycobacterium tuberculosis, *NTM* non-TB mycobacteria, *PTB* pulmonary tuberculosis

## Discussion

Our study has four main findings, namely that (1) bronchopneumonia was the most prevalent lung pathology, (2) comorbidity between non-communicable and communicable diseases was found in 80 % of children, (3) tuberculosis was detected in 8 % of mortalities, with 9/10 cases being undiagnosed and untreated ante mortem, and (4) results of molecular analysis found evidence for MTB in 7/10 histopathologically confirmed TB cases.

The results should be viewed in terms of several limitations. Due to the generic social and resource limitations of conducting full autopsies in any geographical setting [11], our necropsy study had its focus on the chest cavity and was limited by the small sample size and the likelihood of the recruited children being older and male. These biases are difficult to avoid on a consenting autopsy study within a community that is broadly culturally opposed to mutilation of the deceased [10]. In addition to slightly lower reservations among families over consenting for autopsy on males and older children, the recruiting clinical officer (CC) felt that families of lower socioeconomic status were more likely to consent, consistent with our recent adult autopsy study [14], but we did not collect any socioeconomic indicators such as maternal education status. Finally, the study was undertaken at a referral centre and so does not include childhood deaths within the community. It should be noted, however, that within Lusaka, referral systems are well established and most childhood deaths occur at UTH. The findings of this study should be interpreted in light of these limitations.

The high prevalence of bacterial lung infections is alarming, as these are supposedly treatable with the range of antibiotics available at UTH, and more broadly within Zambia and regionally. In addition, two thirds of pneumonia cases were not diagnosed ante mortem. These deaths could have been influenced by increasing levels of antibiotic resistance in common community acquired pneumonia pathogens such as *Haemophilius influenzae* and *Streptococcus pneumoniae* [18]. Whilst susceptibility of these two community acquired pneumonia pathogens to front line antibiotics, such as Ampicillin and Amoxicillin, remains quite high in Africa (70–90 %) [18], a mortality study naturally selects for severe infections that are more likely to be drug resistant, present late or be complicated by comorbidities. In the absence of full post mortem we could not determine the relative contribution of lung pathology with histopathological findings from other tissues, but comparison of lung pathology findings with clinical data identified possible comorbidity in up to 80 % of cases. For a minority of cases with severe central nervous system infections or congenital heart disease, the observed lung pathology may have been secondary, but for the overwhelming number of cases, including malnourished children, the study pathologists (AS and VM) considered the lung pathology observed to be the likely primary cause of death. The observed high levels of comorbidity, in particular with malnutrition, are consistent with the high levels of malnutrition in Zambia [19]. Severely malnourished children are at increased risk of respiratory infections and associated mortality [20].

Interstitial pneumonitis is defined as a thickening of the interstitium, which damages alveoli architecture and function [21]. It can be caused by bacterial, viral or fungal lung infections, and also has a range of non-infectious aetiologies such as air pollutants. In poor communities in urban Zambia, most cooking is done on charcoal [22]. As the second most prevalent cause of fatal lung pathology, observed in 17 % (20/121) of cases, the aetiology of interstitial pneumonitis in African children requires further definition, possibly through immunocytochemistry or next generation sequencing of tissue specimens. It is challenging to establish a definitive diagnosis ante mortem, with chest x-ray manifestations having considerable overlap with other lung diseases such as TB [7].

Histopathologically confirmed TB (observation of granulomas, caseous necrosis and Langhans giant cells) was detected in 8 % (10/121) of cases. Molecular analysis confirmed the presence of *M.tb* DNA in 7/10 cases, and evidenced possible NTM infection in two cases. A recent national study determined the prevalence of symptomatic NTM infection in Zambian adults to be three-fold higher than the national TB prevalence [23], which suggests NTM infections could also be a significant cause of disease in children as reviewed [24]. *M.tb* and NTM specific DNA was also commonly detected in the absence of histopathological evidence of TB infection, possibly indicative of latent infections, including three cases with rifampicin resistance or comorbidity where less severe TB/NTM infection was secondary to bronchopneumonia or other causes of death.

These findings seem at odds with Global Burden of Disease estimates, which do not consider TB as a notable cause of death in African children [25]. However, they are consistent with the views of other leading commentators [26–28] and our previous paediatric autopsy study (conducted between 1997–2000) which confirmed TB as a cause of death in 20 % (54/264) of cases, with the point prevalence being higher among HIV-uninfected children (26 %) than among HIV-infected children (18 %) [12]. Similarly in this study, we found TB in both HIV-infected and uninfected children. In Zambia, childhood TB notification rates have fallen over the last decade [29], but TB clearly remains an important cause of death. Importantly, only 10 % (1/10) of TB cases were on TB treatment when they died, illustrating how poor TB diagnostic services are for children at UTH. Despite significant improvements in HIV prevention, diagnosis and treatment over the last decade [30], it is the slow pace of improvements in TB diagnostic tools and services for children [31] that has allowed TB to persist as a significant cause of death in children. A recent modelling study suggested that, for 15 high burden countries, only 35 % of incident paediatric TB cases are notified globally [26].

Malnutrition was observed in 80 % of histopathologically confirmed TB cases. The mortality rate on the malnutrition ward within the department of paediatrics and child health at UTH is 18 %, second highest only to the neonatal intensive care unit (30–50 %) [32]. More active surveillance of TB and bacterial pneumonia may be justified in malnourished paediatric admissions, and among malnourished children with TB contact, attending community clinics, at high risk of referral to UTH. There is maybe reservation to conduct invasive sampling in severely malnourished children, as it may be unpleasant for the patient. As they are on antibiotics to cover bacterial lung or gut infections and PCP, the main focus is on addressing their nutritional needs. This might mean that TB or viral respiratory infections are overlooked. The ideal specimen for TB diagnosis in young children is induced sputum [33] but spent feeding tubes could be considered as a non-additionally invasive source of gastric aspirate for TB analysis in severely ill malnourished children [16].

The point prevalence of CMV pneumonia (7 %) and PCP (5 %) were both lower than previously reported [12], contrary to our previous study where, with a larger sample, we saw a strong association with HIV infection (CMV = 22 %, PCP = 29 %). The previous study was undertaken prior to paediatric ART roll-out in Zambia and prior to the implementation of cotrimoxazole prophylaxis in HIV-infected children with pneumonia [12], which may have impacted on reducing CMV and PCP-associated deaths in HIV-infected children. The previous study also included only respiratory mortalities, had a lower median age, and used both immunocytochemistry as well as the observation of classic ‘owls eye’ inclusions to define CMV infection [12]. We have shown, in a recent population-based study [34], that early infant CMV infections are linked with impaired development of Zambian infants and, that among admitted infants, CMV DNAemia is associated with being underweight, meningitis, and HIV infection [35], suggesting CMV is an important determinant of health in African children. CMV pneumonia is currently treated with intravenous Ganciclovir at some centres in South Africa [36–38]. CMV was not considered ante mortem because Ganciclovir is not currently available at UTH, can cause leukopenia and neutropenia [39], and could be harmful in some HIV-infected children with a low CD4. There is a need for controlled trials for anti-CMV drugs in the African paediatric setting [36, 40].

## Conclusions

The high level of discrepancy between clinical diagnoses and post mortem findings is alarming but is consistent with previous adult autopsy studies undertaken in Africa [14, 41–45], and in many cases is suggestive that patients were not receiving optimal care. Whilst some undiagnosed infections may be captured by empirical broad-spectrum antibiotic therapy, important pathogens such as TB and CMV were undiagnosed and untreated in most cases. The high burden of bacterial pneumonia despite the availability of a broad panel of antibiotics raises questions over levels of drug-resistant bacterial lung infections among paediatric admissions and management of children with comorbidities. Interstitial pneumonitis is a common cause of death and its precise aetiology in African children requires further definition. TB is not considered a significant cause of death among children by the broader global health community [1, 25], but our demonstration of the importance of TB as a cause of death in Zambian children, first in 2002 [12] and now again over a decade later, supported by molecular analysis, suggests that more intensified TB case finding among children is needed at all levels of healthcare, with a particular focus on malnourished children. UTH and other hospitals serving high TB burden urban communities in sub-Saharan Africa might want to consider piloting intensified TB case finding among malnourished paediatric admissions.
